# Intracellular Delivery of Itaconate by Metal–Organic Framework-Anchored Hydrogel Microspheres for Osteoarthritis Therapy

**DOI:** 10.3390/pharmaceutics15030724

**Published:** 2023-02-22

**Authors:** Han Yu, Peng Ren, Xuekang Pan, Xinyu Zhang, Jun Ma, Jiayi Chen, Jian Sheng, Huanhuan Luo, Huigen Lu, Gang Chen

**Affiliations:** 1 Jiaxing University Master Degree Cultivation Base, Zhejiang Chinese Medical University, Hangzhou 310000, China; 2 Jiaxing Key Laboratory of Basic Research and Clinical Translation on Orthopedic Biomaterials, Department of Orthopaedics, The Second Affiliated Hospital of Jiaxing University, 1518 North Huancheng Road, Jiaxing 314000, China; 3 Graduate School of Bengbu Medical College, Bengbu 233030, China

**Keywords:** itaconate, zeolitic imidazolate framework-8, pH-sensitive, microfluidic, osteoarthritis

## Abstract

Treatment of osteoarthritis (OA) remains a significant clinical challenge. Itaconate (IA), an emerging regulator of intracellular inflammation and oxidative stress, may potentially be harnessed to treat OA. However, the short joint residence time, inefficient drug delivery, and cell-impermeable property of IA can seriously hamper the clinical translation. Herein, IA-encapsulated zeolitic imidazolate framework-8 (IA-ZIF-8) nanoparticles were self-assembled by zinc ions, 2-methylimidazole, and IA to render them pH-responsive. Subsequently, IA-ZIF-8 nanoparticles were firmly immobilized in hydrogel microspheres via one-step microfluidic technology. It was demonstrated in vitro experiments that IA-ZIF-8-loaded hydrogel microspheres (IA-ZIF-8@HMs) exhibited good anti-inflammatory and anti-oxidative stress effects by releasing pH-responsive nanoparticles into chondrocytes. Importantly, compared with IA-ZIF-8, IA-ZIF-8@HMs showed better performance in the treatment of OA due to their superior performance in sustained release. Thus, such hydrogel microspheres not only hold enormous potential for OA therapy, but also provide a novel avenue for cell-impermeable drugs by constructing appropriate drug delivery systems.

## 1. Introduction

Osteoarthritis (OA), characterized by progressive degeneration of articular cartilage, osteophyte formation, and chronic inflammation, is a painful and highly disabling disease [1]. There are many factors associated with osteoarthritis, including various biochemical, genetic, and mechanical factors [2]. In recent years, it has been reported that chronic inflammation plays an essential role in OA initiation and development [3,4]. Inflammatory mediators, released from cartilage, bone, and synovium, can promote the degradation of the cartilage extracellular matrix in OA progression [5]. Degraded cartilage fragments induce and exacerbate disruption of anti-inflammatory and pro-inflammatory pathways and vice versa [6]. Unfortunately, non-steroidal anti-inflammatory drugs, as the conventional strategy, can attenuate short-term clinical symptoms, but can hardly mitigate the progression of OA. Moreover, they are in high systemic toxicity and inefficient drug delivery without repeated administration [7]. Although several drug delivery platforms have been developed in preclinical studies to overcome these deficiencies, few have been successfully translated into the clinic [8]. Therefore, there is currently unmet medical need for OA therapy to design and optimize new anti-inflammatory drug delivery platforms.

Itaconate (IA) which is regulated by immune-responsive gene 1 is an α,β-unsaturated dicarboxylic acid [9]. Recent work has confirmed IA as a novel and effective regulator of intracellular inflammation and oxidative stress [10]. However, IA can hardly penetrate the cell membrane. In addition, several cell-permeable IA derivatives have been synthesized to address this drawback. For instance, it was reported that the production of inflammatory cytokines (i.e., IL-1β, IL-12p70, and IL-6) was significantly decreased in bone-marrow derived macrophages after pre-treated with a cell-permeable derivative of itaconate, which was designated as dimethyl itaconate [11]. Mills and colleagues reported a new cell-permeable esterified derivative of itaconate (i.e., 4-octyl itaconate), which was characterized by its octylester tail, and they confirmed that 4-octyl itaconate could downregulate the levels of inflammation factors and be protective against lipopolysaccharide-induced lethality in vivo [12]. Nevertheless, these IA derivatives may be insufficient to perfectly mimic endogenous itaconate, while there has not been any evidence to confirm whether the effects observed with itaconate derivatives are itaconate-dependent [13]. The burgeoning field of drug delivery systems may provide an effective approach to increase the concentration of endogenous itaconate.

In recent years, metal-organic frameworks (MOFs) have been extensively studied and considered promising drug carriers for biomedical applications due to their large surface areas, well-defined pore sizes, and tunable functionalities [14,15,16,17]. Zeolitic imidazolate frameworks (ZIFs), a subfamily of MOFs, include many different tetrahedral transition metal ions. For instance, ZIF-4 is constructed by the coordination between zinc ions and imidazole anions, and ZIF-67 is prepared by cobalt ions and 2-methylimidazole [18]. In particular, zeolitic imidazolate framework-8 (ZIF-8), constructed by 2-methylimidazole and zinc ions, is one of the most attractive ZIFs [19]. ZIF-8 offers many advantages, including low cytotoxicity, ideal drug loading, and pH-responsive property [20]. Importantly, due to the facile polymerization with various objects, ZIF-8 is widely applied for the encapsulation of drugs, enzymes, and even nanoparticles to further develop pH-responsive drug delivery systems [18,21,22]. Inspired by this, it is possible that the excellent drug loading, cell-permeable ability, and pH-responsiveness of ZIF-8 may help to get into the chondrocytes and have a responsive release of IA.

Nanoparticles with a small particle size are prematurely cleared in the joint cavity [23]. Combining hydrogel matrix with functional nanoparticles is expected to allow the composites to exert better therapeutic effects [24], meeting the vision of nanoarchitectonics [25]. Fortunately, microfluidic technology has the advantages of low reagent consumption, fewer samples, and favorable analytical performance, which have greatly expanded its application in drug delivery [26]. Gelatin methacrylate (GelMA) hydrogel microspheres (HMs) prepared by one-step microfluidic technology have been widely used as an excellent biocompatibility platform for tissue engineering [27]. In addition, the injectable, highly dispersed, and size-uniform HMs synthesized by microfluidic technology could suspend in the synovial fluid and retain for a long time due to their micron size as Yang et al. reported [28]. Consequently, HMs, constructed by microfluidic technology, offer an ideal option as nanoparticle carriers for the sustained release of nanoparticles due to the impediment from photo-crosslinkable hydrogel network [29].

**Scheme 1:**
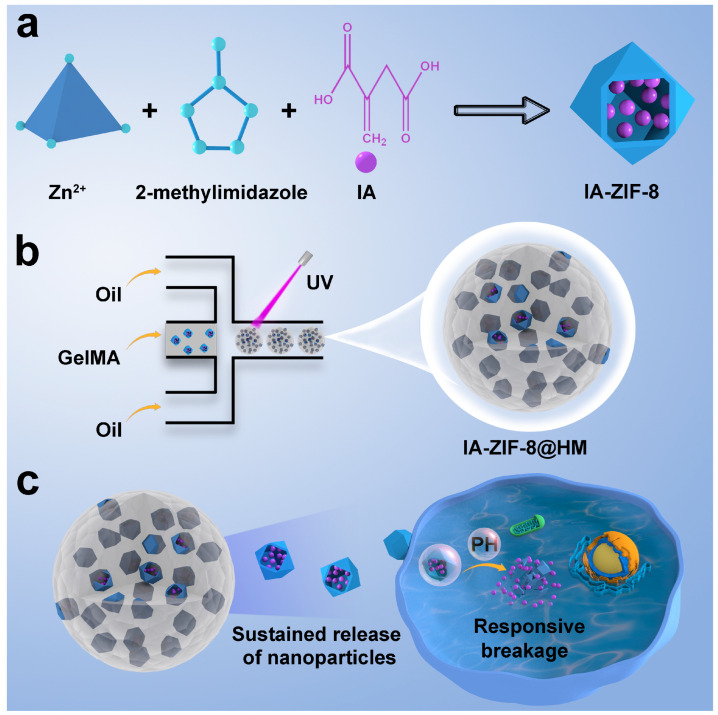
Illustration of nanoparticle assembly, nanoparticle-encapsulated hydrogel microsphere fabrication, and osteoarthritis (OA) treatment. (**a**) The synthesis of the itaconate (IA)-encapsulated zeolitic imidazolate framework-8 (IA-ZIF-8) nanoparticles. (**b**) The fabrication of IA-ZIF-8-loaded hydrogel microspheres (IA-ZIF-8@HMs) by one-step microfluidic technology under ultraviolet (UV) light. (**c**) The design of IA-ZIF-8@HMs for treating OA.

Herein, as illustrated in Scheme 1, IA-loaded ZIF-8 (IA-ZIF-8) nanoparticles were fabricated by the self-assembly of zinc ions, 2-methylimidazole, and IA. Then, pH-responsive IA-ZIF-8 nanoparticles were incorporated into hydrogel matrix as secondary structures using microfluidic technology, followed by photo-crosslinkable processes under ultraviolet (UV) light. The monodisperse HMs which were encapsulated with IA-ZIF-8 nanoparticles (IA-ZIF-8@HMs) could suspend in the synovial fluid and sustained release nanoparticles. Importantly, IA-ZIF-8 nanoparticles diffused from HMs were effectively trafficked into intracellular lysosomes and decomposed under the acidic environment of lysosomes [30], correspondingly inducing the pH-responsive release of IA, which could inhibit oxidative injury and suppress inflammatory response. In conclusion, this drug delivery platform might provide a new and valid strategy for long-term and effective OA therapy.

## 2. Materials and Methods

### 2.1. Materials

Zinc nitrate hexahydrate, 2-methylimidazole, itaconate (IA), methanol, gelatin, phosphate-buffered saline (PBS), methacrylic anhydride, Span 80, and mineral oil were purchased from Macklin Company, Shanghai, China. Microfluidic droplet chips were obtained from MesoBioSystem Company (Wuhan, China). The pressure control pump was purchased from Harvard Apparatus, Holliston, MA, USA.

### 2.2. Preparation of Nanoparticles

ZIF-8 and IA-ZIF-8 were prepared according to the method reported previously [31,32,33]. In detail, 10 mg of IA was dissolved in 4 mL methanol under ultrasonication, while 200 mg of zinc nitrate hexahydrate was dissolved in 0.8 mL of methanol. Next, the above solution was mixed and stirred for 5 min. Then, 10 mL of 2-methylimidazole solution (44 mg/mL), prepared in methanol, was added slowly into the mixture and stirred for 15 min. Finally, milk-white IA-ZIF-8 nanoparticles were obtained by centrifugation (13,000 rpm) and washed with a mixture of methanol and H_2_O, while the solid IA-ZIF-8 nanoparticles were dried under vacuum. As for ZIF-8, zinc nitrate hexahydrate solution was mixed with methanol, and the other procedures were the same as those of IA-ZIF-8 fabrication.

### 2.3. Preparation of GelMA

As reported previously [34], 20 g of gelatin was first dispersed into 200 mL PBS and subsequently 16 mL of methacrylic anhydride was added dropwise at 60 °C under stirring for 2 h. Afterward, 200 mL of PBS was added into the mixture to terminate the reaction. The solution was dialyzed using a dialysis bag, and purified for 3 days. Finally, the lyophilized GelMA was collected after being stored at −80 °C and freeze-drying.

### 2.4. Preparations of HM and IA-ZIF-8@HM

The preparation methods of HM and IA-ZIF-8@HM were with reference to our previous study [35]. In brief, 15 mg of 2-hydroxy-4′-(2-hydroxyethoxy)-2-methylpropiophenone and 150 mg of solid GelMA were dissolved into 3 mL of PBS to be served as the aqueous phase. For the preparation of the oil phase, 5% Span 80 was added into the mineral oil. Next, both the oil phase and aqueous phase were introduced into a microfluidic droplet chip via a pump to synthesize dispersive GelMA droplets, followed by photo-crosslinkable processes under UV light for 4 min. In addition, the flow rate ratio of the oil phase to the water phase was controlled in 2:1. For further purification, these HMs were washed repeatedly with 75% ethanol. Then, HMs were placed at −80 °C overnight and lyophilized. IA-ZIF-8@HM was fabricated by adding IA-ZIF-8 nanoparticles to the aqueous phase under ultrasonication, and the other procedures were consistent with those of HM fabrication.

### 2.5. Characterization

The morphology and structure of nanoparticles (i.e., ZIF-8 and IA-ZIF-8) were characterized by a transmission electron microscope (TEM, Tecnai-G20). The size distribution of nanoparticles was investigated using a Zetasizer Nano ZSE (Malvern). X-ray diffraction (XRD) characterization was performed via an X-ray diffractometer (D8 ADVANCE). The pH-responsive behavior of IA-ZIF-8 was carried out in different buffers of pH 7.4 and pH 5.4, while the changes in morphology and size distribution were measured, respectively, with a TEM and Zetasizer Nano ZSE. Scanning electron microscopy (SEM, HITACHI SU8010) and bright-field microscope were used to detect the morphologies of HMs and IA-ZIF-8@HMs. The functional groups in different samples were determined by a Fourier transform infrared (FTIR, Nicolet iS5) spectrometer at a wavelength from 400 cm^−1^ to 4000 cm^−1^.

### 2.6. Biocompatibility of Biomaterials

ATDC5 cells, a kind of mouse chondrogenic cells [36,37], were incubated with different materials (i.e., ZIF-8, IA-ZIF-8, and IA-ZIF-8@HM) in 6-well plates at a density of 1 × 10^6^ cells/well. On days 1, 2, and 3, the cells were processed using Live/Dead cell staining dye for 30 min and then imaged by a fluorescent microscope.

The cells were co-cultured with the above-described materials in 96-well plates with a density of 5 × 10^3^ cells/well to further measure the cell viability. On days 1, 2, and 3, the MTT assay was used to detect the OD value of each group. Moreover, the concentration of nanoparticles that was suitable for cell growth was detected by MTT assay.

### 2.7. Anti-Oxidant Activity of the IA-ZIF-8@HM

The anti-oxidative stress effects of composites were evaluated as previously reported [38,39]. Briefly, the cells were seeded in a 96-well plate at a density of 5 × 10^3^ cells/well overnight. Then, the cells were pretreated with the above-described materials for 4 h and incubated with H_2_O_2_ for 36 h. The cell viability was analyzed via MTT assay.

### 2.8. Intracellular Uptake of Biomaterials

For fluorescence localization, indocyanine green (ICG) was applied to monitor the endocytosis of the nanocomposites. ZIF-8 nanoparticles encapsulated with ICG and IA (ICG/IA-ZIF-8) were fabricated using the steps described above, followed by being anchored into HMs (here designated as ICG/IA-ZIF-8@HMs). Subsequently, cells were incubated with ICG/IA-ZIF-8@HMs on cell slides in 6-well plates. At different time points, cells were washed with PBS three times and subsequently incubated with Hoechst 33342 and Lysotracker Green DND 26, respectively. Finally, the slides were observed under a fluorescent microscope.

### 2.9. Determination of Inflammation Factors

As reported previously [40], IL-1β was used to simulate the inflammatory microenvironment in OA progression. The cells were seeded in a 6-well plate with a density of 1 × 10^6^ cells/well overnight, followed by IL-1β (10 ng/mL) stimulation and incubation with the above-described materials. The levels of inflammation factors (TNF-α and IL-6) in the supernatants were examined by using enzyme-linked immunosorbent assay (ELISA) kits.

### 2.10. In Vivo Therapeutic Efficacy Evaluation

After adapting to laboratory conditions, male Sprague-Dawley (SD) rats (8 weeks old, 271.3 ± 15.3 g) were used to construct OA model in which OA was induced by mono-iodoacetic acid (MIA, 2.5 mg) [41,42]. After modeling, the OA rats in different groups (n = 3) began to be, respectively, intra-articular injected with 50 µL of PBS, ZIF-8 nanoparticles, IA-ZIF-8 nanoparticles, and IA-ZIF-8@HMs every week. The concentrations of nanoparticles in ZIF-8 and IA-ZIF-8 groups were 1 mg/mL, and the concentration of IA-ZIF-8 nanoparticles in IA-ZIF-8@HM group was adjusted to 1 mg/mL. After 5 weeks of treatment, different groups of rats were subjected to X-ray radiography analysis, followed by the measurement of the relative articular space width. Furthermore, the joint samples were harvested for paraffin embedding, followed by being sliced into sections for further H&E staining and safranin O-fast green staining.

### 2.11. Statistical Analysis

All data were expressed as means ± SD and analyzed with Student’s t test via SPSS 25.0 software. Differences of * *p* < 0.05, ** *p* < 0.01, and *** *p* < 0.001 were considered significant.

## 3. Results and Discussion

### 3.1. Synthesis and Characterization of Biomaterials

**Figure 1:**
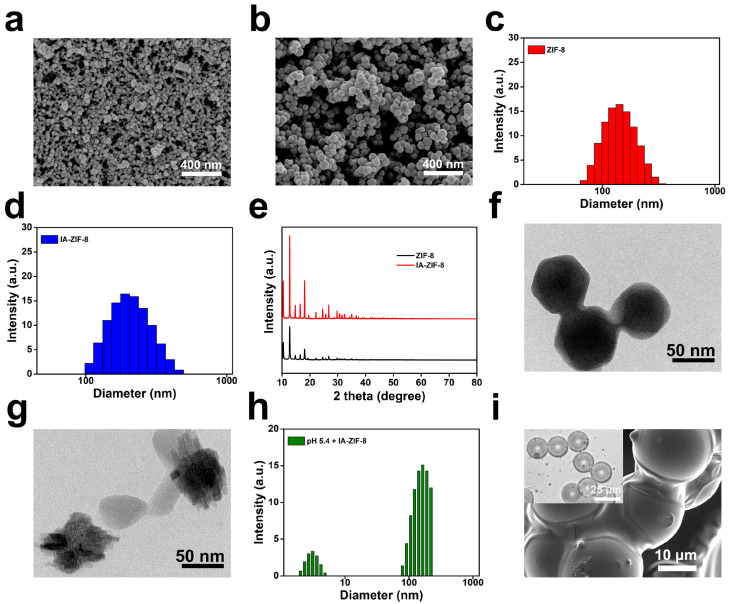
Physical characterizations of nanoparticles and composites. (**a**,**b**) Scanning electron microscopy (SEM) image of zeolitic imidazolate framework-8 (ZIF-8) and IA-ZIF-8. (**c**,**d**) Size distribution of ZIF-8 and IA-ZIF-8. (**e**) X-ray diffraction (XRD) patterns of ZIF-8 and IA-ZIF-8. (**f**,**g**) Transmission electron microscope (TEM) image of IA-ZIF-8 in different buffers of pH 7.4 (**f**) and pH 5.4 (**g**). (**h**) Size distribution of IA-ZIF-8 in buffer of pH 5.4. (**i**) Microscopic image (scale bar: 25 μm) and SEM image (scale bar: 10 μm) of IA-ZIF-8@HMs.

Nanoparticles in this study were fabricated through self-assembling method. The TEM images in Figure 1a,b showed that both ZIF-8 and IA-ZIF-8 nanoparticles were successfully synthesized with highly dispersed, crack-free, and polyhedral morphologies, while the particle sizes of them were 43.62 ± 3.59 nm and 79.57 ± 7.89 nm, respectively. The changes in particle size might be attributed to the encapsulation of cell-impermeable IA, and the nanoscale of IA-ZIF-8 nanoparticles was the optimal particle size for endocytosis as previously reported [43]. Furthermore, the dynamic light scattering (DLS) data in Figure 1c,d showed that the hydrodynamic sizes of IA-ZIF-8 nanoparticles increased from 143.9 nm to 199.8 nm relative to the non-drug-loaded ZIF-8, while the polydispersity index (PDI) was simultaneously changed from 0.194 to 0.122. The crystal structures of ZIF-8 and IA-ZIF-8 nanoparticles were further detected via XRD measurement. As illustrated in XRD dates, the crystalline structure of IA-ZIF-8 was morphologically analogous to the non-drug-loaded ZIF-8 (Figure 1e), which revealed the stability of the crystalline structure of ZIF-8 during the drug encapsulation process.

To further validate the pH-responsive property of IA-ZIF-8, morphology and DLS measurements were carried out. The structures of IA-ZIF-8 nanoparticles were collapsed in the TEM images (Figure 1f,g and Figure S1a,b), while the hydrodynamic size and PDI were changed after being immersed in PBS at a pH of 5.4 (Figure 1h), indicating the favorable pH-responsive property of IA-ZIF-8. Subsequently, the micron-scale composites were fabricated through one-step microfluidic technology. The microscopic and SEM images presented that the size-uniform, spherical, and smooth HMs were successfully prepared with a diameter of 19.55 ± 0.67 μm (Figure S2a,b), and the outward appearances of IA-ZIF-8@HMs were uneven with the diameter of 20.25 ± 0.43 μm (Figure 1i), indicating both HMs and IA-ZIF-8@HMs might retain in the articular cavity for a long time and sustained release nanoparticles. Then, the encapsulation processes of nanoparticles and micron-scale composites were qualitatively verified via FTIR spectroscopy. As shown in Figure S3, the characteristic peaks of the IA group were unobservable in that of the IA-ZIF-8 group and the characteristic peaks of the IA-ZIF-8 group were consistent with that of the ZIF-8 group, which might imply the successful drug encapsulation process. Moreover, the FTIR spectra of HM and IA-ZIF-8@HM groups were provided for comparison. The characteristic peaks of the IA-ZIF-8@HM group were in good agreement with that of the HM group except for the peak at 1164 cm^−1^ from the IA-ZIF-8 group, which indicated the successful incorporation of nanoparticles into IA-ZIF-8@HMs.

### 3.2. In Vitro Cytotoxicity

**Figure 2:**
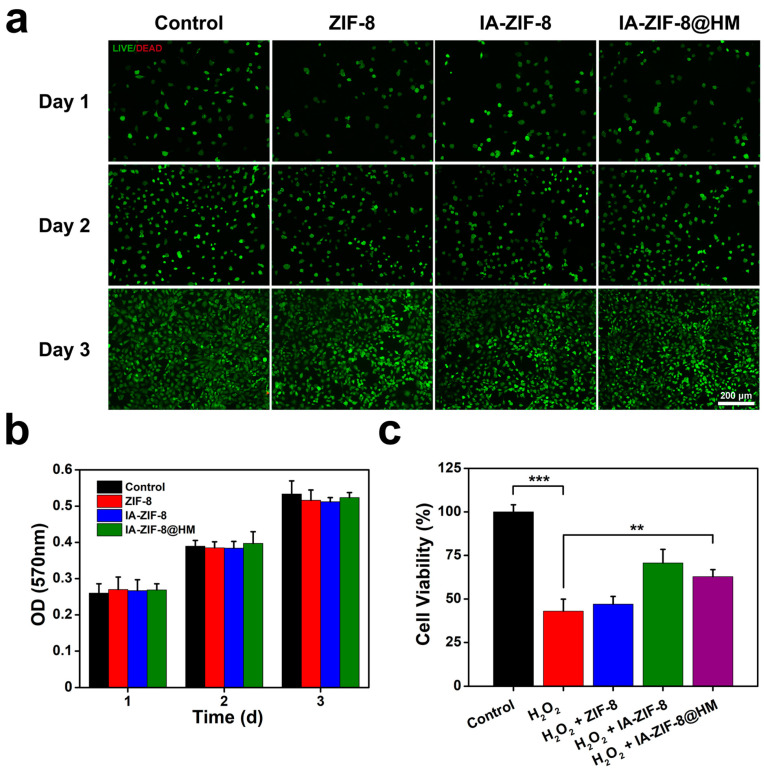
In vitro cytotoxicity and anti-oxidative stress effects of composites. (**a**) The live/dead fluorescence results of control, ZIF-8, IA-ZIF-8, and IA-ZIF-8@HM groups on 1, 2, and 3 days. (**b**) In vitro cytotoxicity of ZIF-8, IA-ZIF-8, and IA-ZIF-8@HM groups on ATDC5 cells detected by MTT assay. (**c**) The results of cell viability about chondrocyte co-cultured with H_2_O_2_, H_2_O_2_ + ZIF-8, H_2_O_2_ + IA-ZIF-8, and H_2_O_2_ + IA-ZIF-8@HM (** *p* < 0.01, *** *p* < 0.001).

The in vitro cytotoxicity of biomaterials on ATDC5 cells should be examined to ensure the feasibility of in vivo experiments. Initially, we ascertained the suitable concentration of nanoparticles for in vitro experiments. The cell viability decreased to 71% or less when the concentration of ZIF-8 was increased to 135 μM and 175 μM, while the cell viability was about 93% when the concentration of ZIF-8 was 85 μM, with almost no cytotoxicity (Figure S4). Therefore, ZIF-8 and IA-ZIF-8 nanoparticles were utilized at 85 μM, and the concentration of IA-ZIF-8 nanoparticles in IA-ZIF-8@HM group was adjusted to 85 µM for the following test. Then, cells were subjected to the Live/Dead and MTT assays after 1, 2, and 3 days of incubation with different biomaterials. Each group of cells basically survived, and the cell density increased gradually throughout 3-day co-cultivation, as displayed in Figure 2a. Additionally, as certified by the MTT assay, the cell number of each group increased continuously, and there was no significant difference between groups. Accordingly, all these biomaterials had good biocompatibility with cells.

### 3.3. The Efficacy of Anti-Oxidative Stress

Oxidative stress can cause progressive matrix degradation in OA by inducing disorders of the catabolic and anabolic pathways [44]. Hence, we investigated whether IA-ZIF-8@HMs could attenuate H_2_O_2_-induced oxidative stress. The proper concentration of H_2_O_2_ was determined by MTT assay to mimic oxidative stress injury in OA. As seen from Figure S5, H_2_O_2_ significantly inhibited cell viability at the concentration of 300 μM, indicating the proper concentration for the anti-oxidative stress experiment. The cell viability of the H_2_O_2_ group was significantly suppressed (Figure 2c). In contrast, a significant increase in cell viability was observed in both IA-ZIF-8 and IA-ZIF-8@HM groups, which might be attributed to the intracellular release of cell-impermeable IA. Taken together, these results indicated that IA-ZIF-8@HMs could inhibit H_2_O_2_-induced oxidative stress, and the cell-impermeable of IA might be trafficked into chondrocytes through the composites in this study.

### 3.4. Cellular Internalization

**Figure 3:**
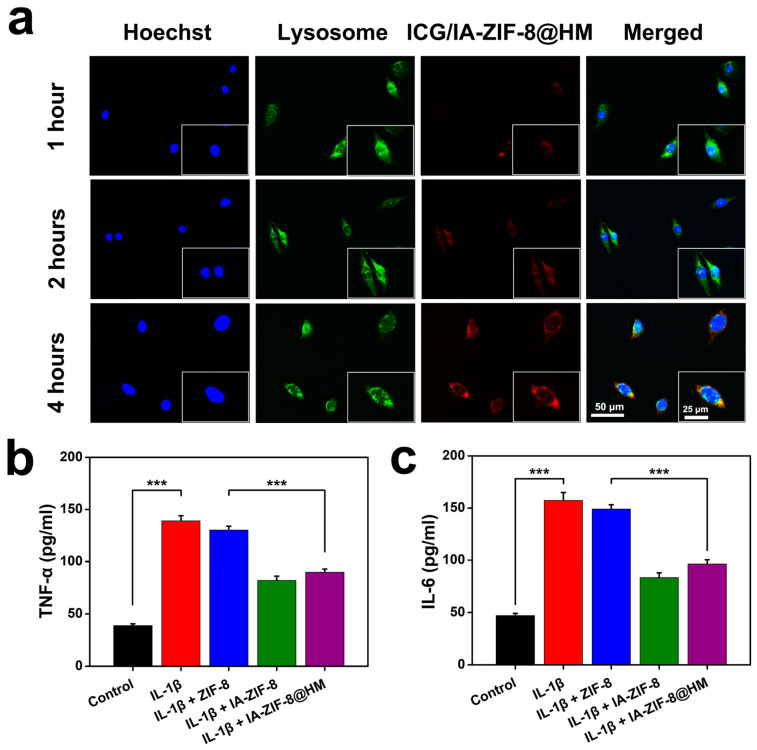
Endocytosis and anti-inflammatory effect of composites. (**a**) The fluorescent microscope images of ATDC5 cells co-cultured with ICG/IA-ZIF-8@HM for 1, 2, and 4 h. (**b**,**c**) Enzyme-linked immunosorbent assay (ELISA) analyses of TNF-α (**b**) and IL-6 (**c**) concentration in cell supernatants (*** *p* < 0.001).

To further confirm the successful intracellular transportation of IA, the cellular uptake of the nanocomposite was assessed by fluorescence localization. As shown in Figure 3a, cells were stained with Hoechst 33342 (blue) in nucleus and Lysotracker Green DND 26 (green) in lysosome. The intensity of red fluorescence, related to the number of intracellular nanocomposites, was initially weak at 1 h and gradually enhanced with co-cultured time. In addition, the red fluorescence of ICG-loaded in nanocomposites matched well with the green fluorescence. These images showed that nanocomposites diffused from hydrogel matrix were effectively trafficked into chondrocytes, which were conducive to the further release of IA in situ.

### 3.5. Anti-Inflammatory Efficacy

IA has been proposed as an emerging regulator of intracellular inflammation and oxidative stress that results in a promising therapeutic application for inflammatory diseases [9]. We further assessed the anti-inflammatory efficacy of IA-ZIF-8@HMs via ELISA kits. As the ELISA analyses presented (Figure 3b,c), compared with the control group, a significant enhancement in the secretion of inflammatory cytokines (i.e., TNF-α and IL-6) was observed in the IL-1β group, indicating the successful construction of the inflammation microenvironment. After being treated with IA-ZIF-8 nanoparticles and IA-ZIF-8@HMs, the levels of inflammation factors exhibited significant drops. These data suggest that IA-ZIF-8@HMs could effectively suppress inflammation and might have a promising therapeutic effect for OA therapy in vivo.

### 3.6. Therapeutic Effect Test In Vivo

**Figure 4:**
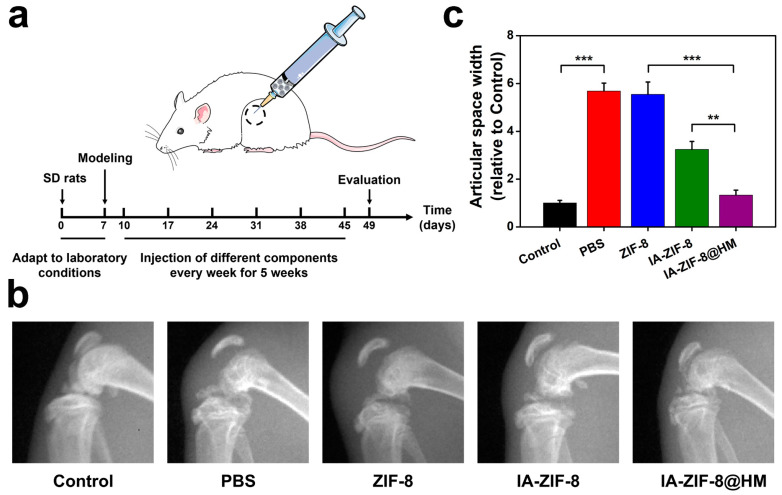
In vivo therapeutic effect of composites. (**a**) Overview of in vivo experiments. (**b**) The X-ray images of different groups after the treatment. (**c**) The relative articular space width of different groups (** *p* < 0.01, *** *p* < 0.001).

After evaluating the anti-oxidative stress and anti-inflammatory efficacy of IA-ZIF-8@HMs in vitro studies, the therapeutic effect in vivo was further investigated. Intraarticular injection of MIA was applied to establish the OA model in this study, because of its less intrusion, simplicity, and good reproducibility [42]. As demonstrated in the overview of in vivo experiments in Figure 4a, different groups of rats were locally injected with different components every week for 5 weeks, followed by in vivo tests. The articular cartilage of PBS and ZIF-8 groups of rats was seriously damaged and the relative articular space widths of them were significantly increased compared with the control group (Figure 4b,c), indicating the considerably progressed OA. In contrast, IA-ZIF-8 and IA-ZIF-8@HM groups showed significant improvement in the morphology of rat knee joints, suggesting the inhibition effects of IA-ZIF-8 nanoparticles and IA-ZIF-8@HMs on OA progress. Moreover, compared with the IA-ZIF-8 group, the relative articular space width of IA-ZIF-8@HM group was significantly decreased, which might be attributed to the sustained release of nanoparticles from hydrogel for long-term therapeutic effect.

**Figure 5:**
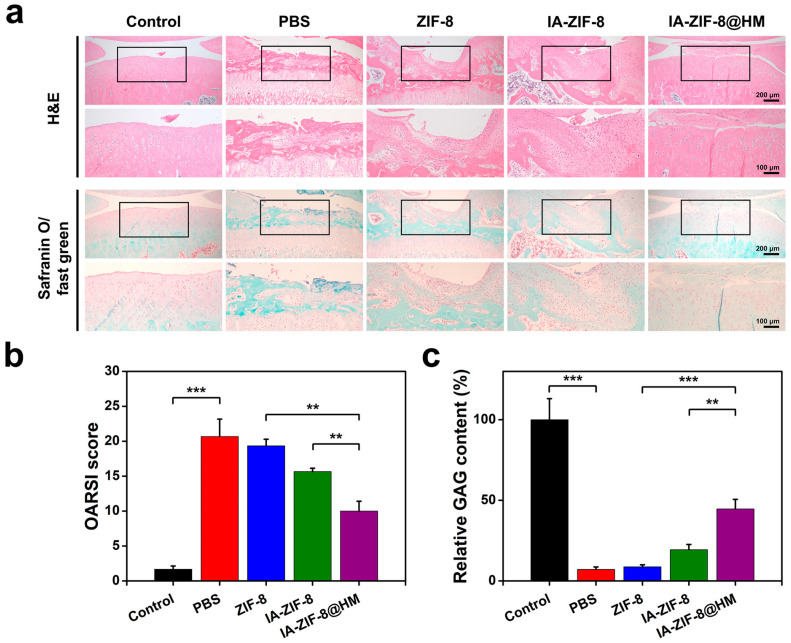
IA-ZIF-8@HM relieved the progression of OA in vivo experiments. (**a**) Histopathological images of each group after the treatment. (**b**) OARSI scores of articular cartilage for different groups after the treatment (** *p* < 0.01, *** *p* < 0.001). (**c**) Relative glycosaminoglycan (GAG) content for different groups after the treatment (** *p* < 0.01, *** *p* < 0.001).

In addition to radiographic assessment, we used H&E staining and safranin O-fast green staining to investigate joint samples for histopathological evaluation. As shown in the histopathological images presented in Figure 5a, in comparison with the control group, corrosion cracks and deformation of articular surface were observed in PBS and ZIF-8 groups. Notably, compared with the IA-ZIF-8 group, the IA-ZIF-8@HM group exhibited significant improvements in characteristics of chondrocytes, morphology changes in articular surface, and matrix staining, proving again the advantages of combining hydrogel matrix with functional nanoparticles. Consistent with the above results, the IA-ZIF-8@HM group showed the lowest value of OARSI score and the best performance in reducing the degradation of cartilage matrix, among all the OA models. In summary, IA-ZIF-8@HMs could effectively ameliorate the progression of OA in vivo, suggesting a valid strategy for treating OA.

Herein, we prepared an infusion pump-like drug delivery platform with pH-responsive secondary structures to treat OA. Different from other studies to synthesize suitable substitutes for itaconate [39,45], infusion pump-like IA-ZIF-8@HMs could suspend in the joint cavity and achieve a sustained release of pH-responsive nanoparticles to promote the intracellular transportation of exogenous IA, realizing anti-oxidative stress and anti-inflammatory efficacy. Moreover, due to the fact that IA can be produced endogenously [12], IA-ZIF-8@HMs might show fewer toxicity issues and superior performance in clinical translation compared with other drug delivery platforms [28,35,41]. However, our studies still contain some limitations; for example, little was done to explore the underlying mechanism associated with the therapeutic effects of exogenous IA.

## 4. Conclusions

In the present study, we innovatively constructed the injectable and monodisperse hydrogel microspheres (i.e., IA-ZIF-8@HMs) to encapsulate nanosized and cell-permeable secondary structures by microfluidic technology. Briefly, IA-ZIF-8@HMs could achieve a sustained release behavior due to the impediment from hydrogel matrix. In addition, the pH-responsive IA-ZIF-8 nanoparticles released from the HMs were trafficked into intracellular lysosomes, followed by the depolymerization. OA rats were used to further verify the therapeutic effect of IA-ZIF-8@HMs. The in vitro experiments revealed the favorable anti-oxidative stress and anti-inflammatory efficacy of IA-ZIF-8@HMs. Moreover, in vivo experiments indicated the long-term and valid therapeutic effect of IA-ZIF-8@HMs. In conclusion, IA-ZIF-8@HMs might be a promising candidate in drug delivery for osteoarthritis treatment and other biomedical applications.

## Data Availability

Not applicable.

## Supplementary Materials

The following supporting information can be downloaded at: https://www.mdpi.com/article/10.3390/pharmaceutics15030724/s1, Figure S1 TEM image of IA-ZIF-8; Figure S2: Physical characterizations of HMs; Figure S3: Fourier transform infrared (FTIR) spectra of different samples; Figure S4: Effects of different concentrations of ZIF-8 nanoparticles on the viability of ATDC5 cells; Figure S5: Effects of different concentrations of H_2_O_2_ on the viability of ATDC5 cells.
