# Supplementary material for: Intracellular Delivery of Itaconate by Metal–Organic Framework-Anchored Hydrogel Microspheres for Osteoarthritis Therapy
Source: Pharmaceutics. 2023 Feb 22;15(3):724. doi: 10.3390/pharmaceutics15030724 (PMC10051475; doi:10.3390/pharmaceutics15030724)
Supplement: Supplementary file 1 [file pharmaceutics-15-00724-s001.zip › pharmaceutics-2123214-supplementary.pdf]

Supporting Information

Intracellular Delivery of Itaconate by Metal-Organic Framework-Anchored Hydrogel Microspheres for Osteoarthritis Therapy

Han Yu [1,2,†], Peng Ren [2,3,†], Xuekang Pan [2,†], Xinyu Zhang [2,3], Jun Ma [1,2], Jiayi Chen [2], Jian Sheng [2], Huanhuan Luo [2,*], Huigen Lu [2], and Gang Chen [2,*]

[1] Jiaxing University Master Degree Cultivation Base, Zhejiang Chinese Medical University, Hangzhou 310000, China.

[2] Department of Orthopaedics, Jiaxing Key Laboratory of Basic Research and Clinical Translation on Orthopedic Biomaterials, The Second Affiliated Hospital of Jiaxing University, 1518 North Huancheng Road, Jiaxing 314000, P. R. China.

[3] Graduate School of Bengbu Medical College, Bengbu 233030, China

[†] These authors contributed equally to this work.

* Corresponding author:

lhh18801544573@163.com (H. Luo)

adcyy@aliyun.com (G. Chen)

Contents

Supplemental Figures

Figure S1. TEM image of IA-ZIF-8 .... S-3

Figure S2. Physical characterizations of HMs S-4

Figure S3. Fourier transform infrared (FTIR) spectra of different samples S-5

Figure S4. Effects of different concentrations of ZIF-8 nanoparticles on the viability of ATDC5 cells S-6

Figure S5. Effects of different concentrations of H_2O_2 on the viability of ATDC5 cells S-7

Supplemental Figures

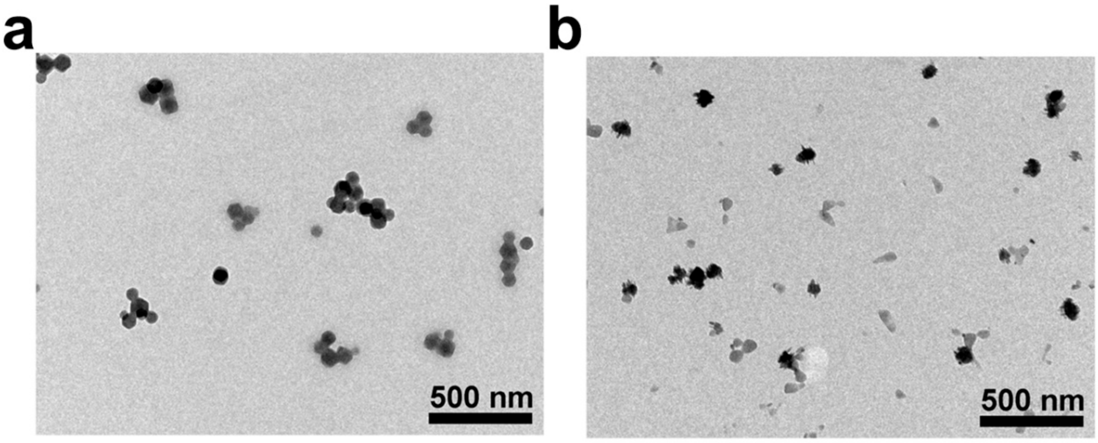

Figure S1. Transmission electron microscope (TEM) image of IA-ZIF-8 in different buffers of pH 7.4 (a) and pH 5.4 (b).

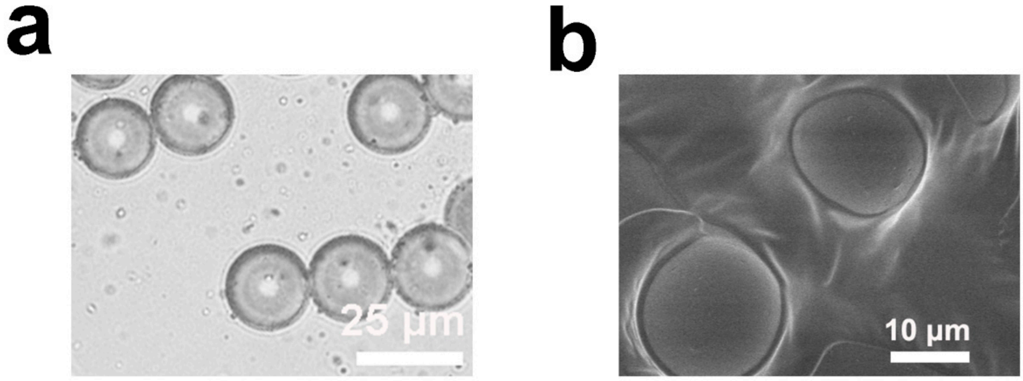

Figure S2. Physical characterizations of HMs. (a) Microscopic images of HMs. (b) Microscopic images of HMs.

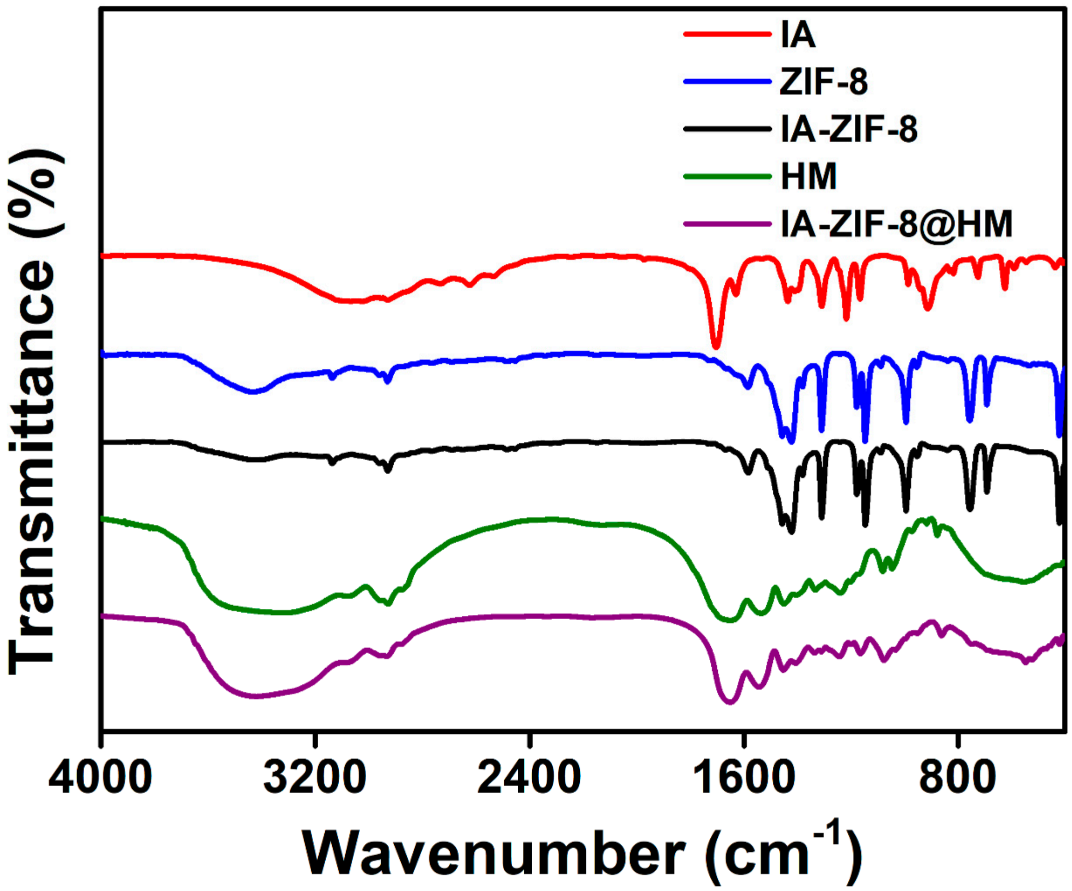

Figure S3. Fourier transform infrared (FTIR) spectra of different samples.

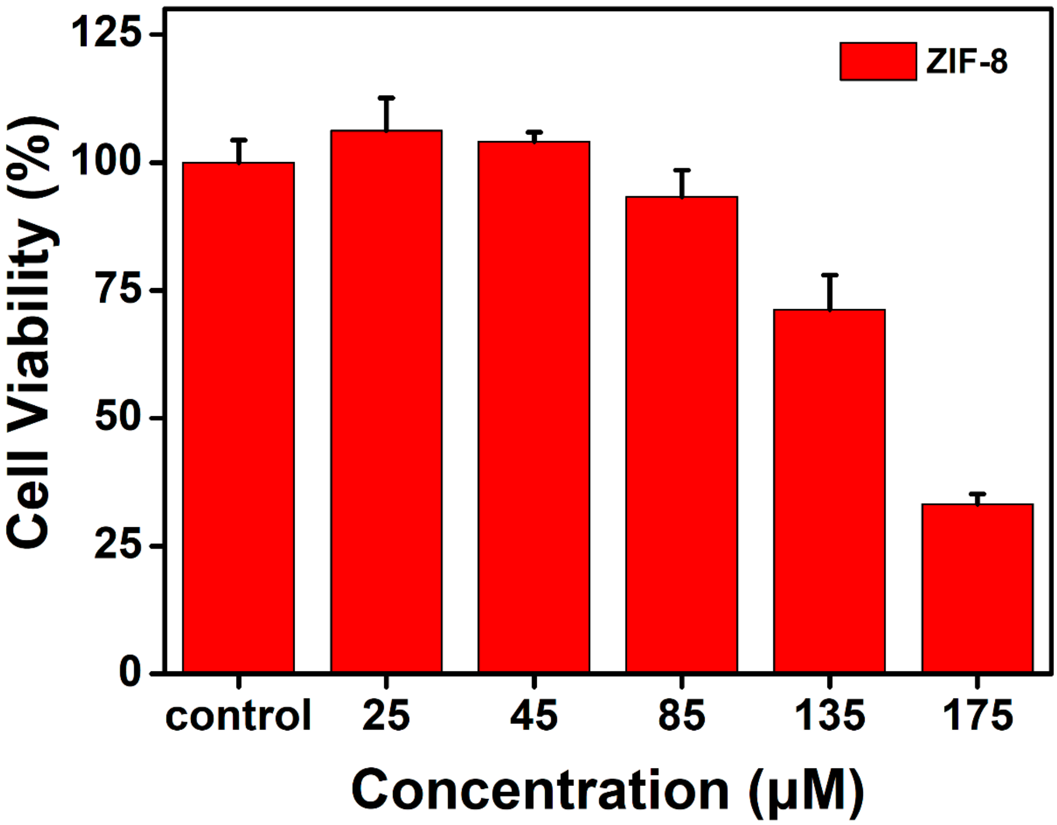

Figure S4. Effects of different concentrations of ZIF-8 nanoparticles on the viability of ATDC5 cells.

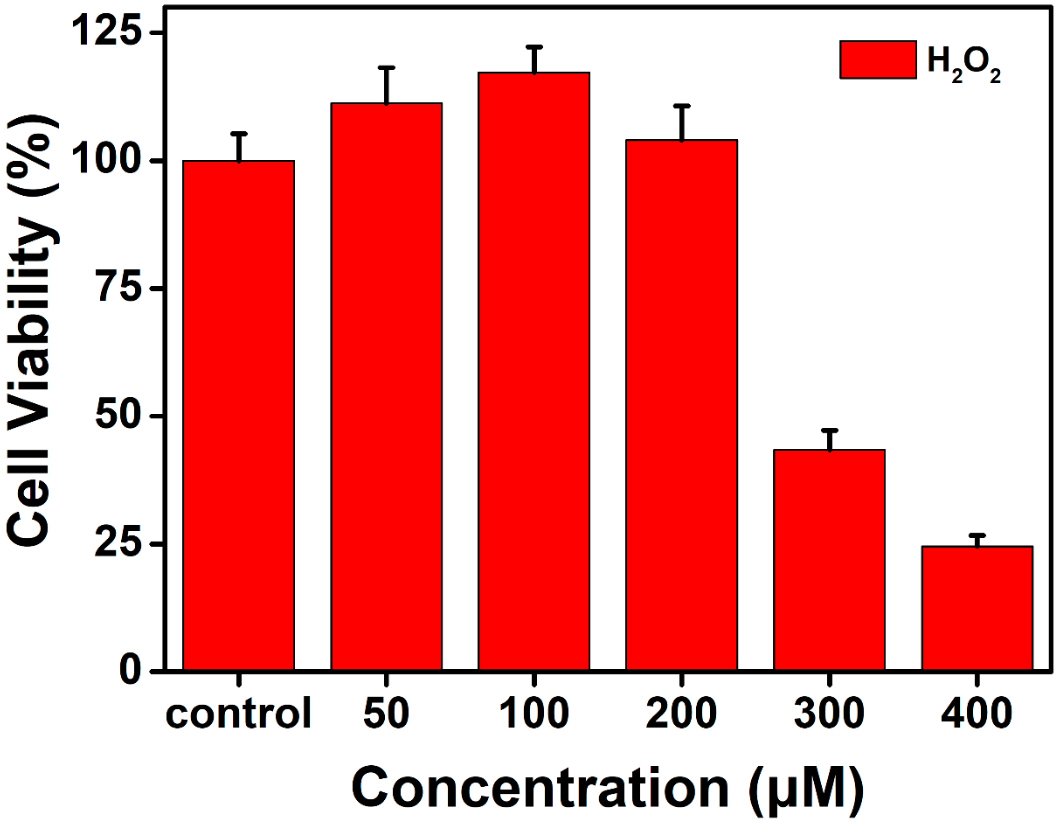

Figure S5. Effects of different concentrations of H_2O_2 on the viability of ATDC5 cells.
